# Potential implications of CYP3A4, CYP3A5 and MDR-1 genetic variants on the efficacy of Lopinavir/Ritonavir (LPV/r) monotherapy in HIV-1 patients

**DOI:** 10.7448/IAS.17.4.19589

**Published:** 2014-11-02

**Authors:** Giulia Berno, Mauro Zaccarelli, Caterina Gori, Massimo Tempestilli, Luigia Pucci, Andrea Antinori, Carlo Federico Perno, Leopoldo Paolo Pucillo, Roberta D'Arrigo

**Affiliations:** 1 Antiviral Drug Monitoring Unit, National Institute for Infectious Disease “L.Spallanzani”, Rome, Italy; 2 Clinical Department, National Institute for Infectious Disease “L.Spallanzani”, Rome, Italy; 3 Clinical Biochemistry and Pharmacology Laboratory, National Institute for Infectious Disease “L.Spallanzani”, Rome, Italy; 4 Department of Experimental Medicine and Surgery, University of Rome Tor Vergata, Rome, Italy

## Abstract

**Introduction:**

Several genetic single nucleotide polymorphisms (SNPs) in biotransformation enzymes (CYP3A4, CYP3A5) or transporter proteins (multidrug resistance MDR1 gene product, P-gp) are involved in PI metabolism so that PI pharmacokinetics is characterized by a large inter-individual variability. The aim of this study was: (i) to develop an in-house PCR/direct sequencing, based on DNA purification of full-length CYP3A4 and CYP3A5 genes (SNPs) and MDR1 C3435T variant; (ii) to investigate association of CYP3A4 and CYP3A5 reported or unreported genetic polymorphisms and MDR1-C3435T (CC homozygote, CT heterozygote, TT homozygote) with clinical outcome of HIV-1 infected subjects treated with PI.

**Methods:**

Overall, 39 HIV-1 infected patients receiving boosted Lopinavir (LPV/r) monotherapy after virological suppression were genotyped and analyzed through PCR and direct sequencing of full-length CYP3A4 and CYP3A5 gene sequences [1] and MDR1 gene (C3435T). CD4+T-cell counts and plasma viral load were analyzed before and after LPV/r initiation; LPV/r therapeutic drug monitoring (TDM) was determined at 12-hours.

**Results:**

LPV/r TDM (ng/ml) did not show significant differences among CYP3A4 or CYP3A5 SNPs, although a mean lower level of LPV/r was associated with detection of several SNPs: CYP3A5*3 rs776746; CYP3A5 rs28365088, CYP3A5 rs15524, CYP3A4 rs2687116, and a not already described polymorphism CYP3A4 nt20338. In follow-up analysis, <90% adherence was the main factor associated with virological failure of LPV/r monotherapy (83.3% of failure vs 34.4%, p<0.001 at log-rank test). Adjusting for adherence, the detection of a single CYP3A5*3 rs776746 and CYP3A5 rs15524 SNPs was associated with higher probability of LPV/r monotherapy failure (p<0.01), and in general, detection of any CYP3A5 SNP was associated with failure (26.2% vs 58.3%, p=0.067). No-association with detection of any CYP3A4 SNPs was found. MDR1 TT variants showed significant lower frequency of treatment failure (0.0% vs 47.7%, p=0.026), since non-TT homozygote patient failed LPV/r monotherapy.

**Conclusions:**

Efficacy of PI monotherapy is strongly dependent from patient adherence, but, in adherent patients, genetic factors, such as CYP3A5 and MDR1-C3435T gene variants, may affect the response to treatment, though their role, as well of other genetic variants, need further investigation.

**Figure 1:**
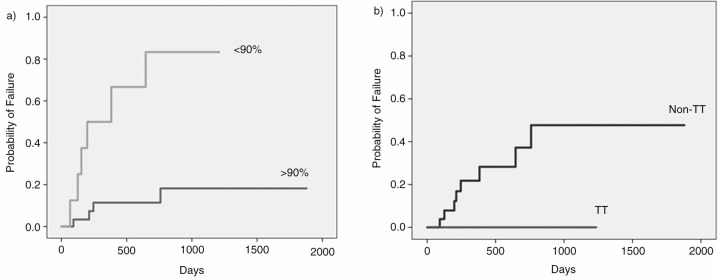
a) Probability of failure of LPV/r monotherapy by treatment adherence; b) Probability of failure of LPV/r monotherapy by MDR1 gene (C3435T).
